# Effect of Self - Care Education on Quality of Life in Patients With Primary Hypertension: Comparing Lecture and Educational Package

**DOI:** 10.5812/nms.11655

**Published:** 2013-12-09

**Authors:** Mohamad Aghajani, Neda Mirbagher Ajorpaz, Mahbube Kafaei Atrian, Zahra Raofi, Fatemeh Abedi, Sajad Naeimi Vartoni, Akbar Soleimani

**Affiliations:** 1 Department of Nursing, Kashan University of Medical Sciences, Kashan, IR Iran

**Keywords:** Lectures, Education, Hypertension, Quality of Life, Self-Care

## Abstract

**Background:**

Hypertension is a dangerous risk factor for public health. It profoundly affects the patients’ quality of life. However, there is lack of agreement on the best method for self-care management in patients with hypertension.

**Objectives:**

This study was conducted to compare the effect of lecture and educational pamphlets on quality of life (QOL) in patients with primary hypertension.

**Patients and Methods:**

A quasi-experimental study was performed on 90 patients with chronic primary hypertension referred to two outpatient clinics in Kashan city. Patients were randomly divided into three groups including lecture group, educational package group, and control group. The participants’ quality of life was measured using the SF-36 questionnaire at the beginning of the study, and two months later. Data was analyzed using ANOVA and Chi-Square tests.

**Results:**

No significant differences were observed between the three groups for demographics characteristics and QOL before the intervention except for marital status. Mean scores of QOL dimensions of the intervention groups were increased at the end of the study, except for the dimension of bodily pain. Tukey post-Hoc test showed that except for general health, the two intervention groups were not significantly different in other dimensions, and significant differences were observed between the control group and the two intervention groups (P < 0.05). At start and the end of the study, the mean differences in the general health dimension in three groups were 2.25 ± 0.1, 0.07 ± 0.01, and -1.70 ± 0.01 respectively. There were significant differences among groups (P = 0.04).

**Conclusions:**

Lecture and educational package can both improve some dimensions of the QOL in patients with hypertension. However, as pamphlets are cheap and easy to use, this method may be used as an effective method for self-care education in health care settings in Iran, where the system is faced with nursing shortage.

## 1. Background 

Hypertension is a dangerous risk factor for public health. It is one of the reasons for disability and death (1). The World Health Organization (WHO) predicted that about 600 million people have high blood pressure and 5.7 million of them die because of this illness and its complications (2, 3). Studies reported its outbreak in Iran. Some researchers have estimated that 19.4% to 22% of Iranians have hypertension (4, 5). In spite of appropriate treatments, management of this problem is undesirable. Azizi et al. showed that 60% of patients with hypertension in Iran have uncontrolled high blood pressure, while they are aware of their illness (4). Hypertension risk factors are diabetes, high level of cholesterol and triglyceride, lack of exercise, bad nutrition and overweigh (6). In addition, hypertension and its complications can significantly reduce the patients’ quality of life (QOL) (7, 8). Previous studies showed that patients with chronic disorders are faced with problems in self-care activities such as management of blood pressure, nutrition, weight control, stress management, and exercise (9). Also it has been shown that self-care training could improve the QOL as a basic method in chronic patients such as patients with heart failure and hypertension (9). Patient education not only leads to a major improvement in risky behaviors such as smoking, but also increases the patients’ stress bearing and physical activity, and then improves patients' QOL (10). It also may prevent some complications, and reduce the need for expensive treatments (11). So it seems that, nurses’ role as trainers can be important in improving QOL in patients with hypertension. There are several ways to deliver health messages. One may be by using the lecture to teach people directly about a particular subject. Someone may use health messages through medium such as books, posters or pamphlets (12). Apparently, different methods of education may have various effects on people’s attitudes and health related behaviors, which consequently affect differently their QOL (13). Hackam et al. conducted an educational program for patients with hypertension, and reported that the program improved the clients’ QOL (14). Gopu et al. have also reported that education through lecture could positively affect the patients’ knowledge, attitudes, and QOL (15). While, Misiaszek et al. have reported that lecturing was more effective than multimedia in improving QOL of patients in Parkinson (16). Moule et al. have shown that educational package was more effective than lecturing methods in improving the QOL (17). The results of an experimental study have also shown that face to face lecturing had positive impacts on some dimensions of QOL of patients with hypertension; while, it did not improve other dimensions (18). Improving the QOL is considered as one of the main objectives of all healthcare and treatment programs (19). Due to the increasing prevalence of hypertension in patients in Iran and around the world, the profound effects of this disorder on the patients’ QOL, and lack of agreement on the best method for patient education, we investigated to find out whether there is any difference between the effects of lecturing and educational pamphlets for the patients’ QOL. 

## 2. Objectives

This study was conducted to compare the effect of lecture and educational pamphlets on QOL in patients with primary hypertension.

## 3. Patients and Methods 

A quasi-experimental study was performed on 90 patients with chronic primary hypertension referred to two outpatient clinics in Golabchi healthcare center and Shahid Beheshti Hospital in Kashan during 2011 - 2012. Inclusion criteria were 35 to 65 year old patients with a medical diagnosis of primary hypertension for more than one year, having a systolic and diastolic blood pressure higher than 140 and 90 mmHg at the beginning of the study, educational level higher than primary school, no previous formal education in blood pressure management, and having similar treatment protocols, having a suitable physical condition, and interest in learning and participating in the study. Exclusion criteria were: receiving extensive information on blood pressure management from other sources, changing the treatment protocol, and getting any significant disorders, and any complication or hypertension crisis.

### 3.1. Sampling

**Figure 1.**
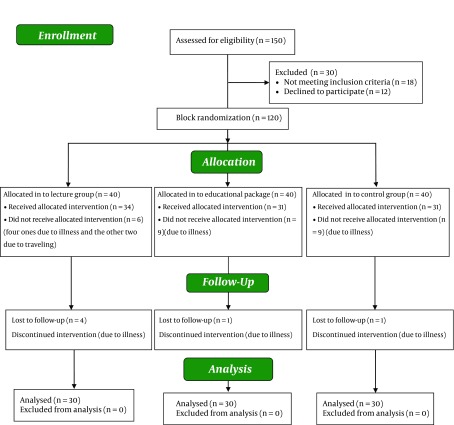
The Sampling Follow-up Diagram

The sample size in each group was determined based on the following assumptions: power = 0.80, α = 0.05, β = 0.20, the minimum expected difference in standard deviation = 3.6, and the minimum expected difference in means to be 2.40 (18). According to the formula, the sample size in each group was 30 members. At first, 150 patients with hypertension were assessed for eligibility. Among those, 18 did not have the inclusion criteria, and 12 persons declined to participate. Therefore, 120 patients were randomly assigned into three groups including lecture, educational package, and control groups by block randomization. Forty patients with hypertension were assigned in each group. Out of the 40 patients assigned to the lecture group, 34 patients were participated and received the allocated intervention, and six did not receive the allocated intervention (four due to illness, and the other two due to traveling). Moreover, four patients lost to follow up during intervention due to myocardial infarction, hypertension crises, appendectomy and travelling. In the second group, 40 patients with hypertension received educational handbook, but 31 patients completed the study. Nine patients did not receive allocated intervention in this group due to hypertension crises, accident, depression, and other illness. Also during intervention, one patient was excluded due to cerebrovascular accident. Also, 40 patients with hypertension were recruited in the control group; however, nine patients did not receive allocated intervention in this group due to hypertension crisis and changing the treatment protocol, persistent headache, heart failure, and diabetes. Also during intervention, one patient was excluded due to cholecystectomy (Figure 1). 

### 3.2. Procedures

In the first group, education was delivered in two sessions using Power Point-enhanced lectures and group discussions. Each session was conducted in two hours by the second author. An invitation letter was sent to each patient several weeks before the educational sessions. Also, the patients were called to remind the educational sessions the day before education. Educational sessions were held in the Faculty of Nursing and Midwifery under supervision of a cardiovascular specialist. The first educational session focused on the diet, exercise, rest and sleeping, stress and anxiety management and social relations. The second session focused on smoking cessation, timely use of drugs, symptoms, and signs of decrease and increase of blood pressure. At the end of the second session, educational handbooks were also offered to the patients to strengthen the educational program. Also the patients' self-care performance was evaluated by weekly telephone calls after education. Each call lasted for five minutes and was focused on the patients’ adherence of the self-care program. The second group only received the self-education handbook, when referred to Golabchi and Beheshty clinics, and were asked to read and follow the self-care program as presented in the handbook. The control group received no education. It was tried to select patients with the same treatment protocol and this process was checked by the cardiovascular specialist who visited all the patients in the two clinics. A two-part instrument was used for data gathering. The first part included four questions regarding the patients’ gender, marriage status, age, and education level. The SF-36 QOL questionnaire was used as the second part. The SF-36 questionnaire was used in Iran before, and its reliability and validity have been approved (Cronbach's alpha between 0.77 - 0.9) (19). SF-36 questionnaire includes eight aspects of physical role, bodily pain, general understanding of health, power and energy, social function, emotional role, and mental health. Each dimension was scored from 0 to 100. Three optional questions with scores of 0, 50, 100, five optional questions with scores of 0, 25, 50, 75, 100, and six optional questions with scores of 0, 20, 40, 60, 80 and 100 were considered in this questionnaire which the higher score showed the better QOL. After explaining the research objectives and obtaining written informed consent from the participants, they completed the demographic data forms and the SF-36 questionnaires at the beginning of the study. Two months after the intervention, all the patients answered the QOL questionnaire again, when the researcher visited them at their own homes. 

### 3.3. Ethical Considerations

The study was approved by the research deputy and the research ethics committee of Kashan University of Medical Sciences. All the participants signed a written consent form before attending the research. The researchers also prepared educational pamphlet for the control group at the end of the study. The researchers observed all ethical issues in accordance with the Helsinki declaration.

### 3.4. Data Analysis

Data analysis was performed using the SPSS software (version 14). Mean score and standard deviation were calculated. Chi-square and ANOVA tests were used to compare nominal variables between the three groups. Analysis of variance (ANOVA) was also used to compare the statistical difference between the mean differences of QOL dimensions of the three groups. For this purpose the differences between the mean QOL dimensions at the beginning of study, and the mean QOL dimensions at the end of study were calculated. Tukey's test was performed to compare the three groups. A P < 0.05 was considered to be statistically significant in all testes. 

## 4. Results

In total, 60% of lecture group, 53.3% of educational package group, and 43.3% of the control group were female (P = 0.01). Before intervention no significant differences were observed between the three groups regarding QOL and demographics characteristics except for marital status (Table 1). 

**Table 1.** Demographic Characteristics of the Study Groups

Variable	Lecture Group	Educational Package Group	Control Group	Chi-Square	P value
**Gender, No.(%)**					
Female	18 (60)	16 (53.3)	13 (43.3)	3.51	0.1
Male	12 (40)	14 (46.6)	17 (56.6)		
**Marital status, No. (%)**					
Married	25 (83.3)	27 (90)	23 (76.6)	2.86	0.05
Single	5 (16.6)	3 (10)	7 (23.3)		
**Age group, No. (%), y**					
35 - 45	6 (20)	9 (30)	8 (26.6)	3.11	0.28
45 - 55	13 (43.3)	10 (33.3)	14 (46.6)		
55 - 65	11 (36.3)	11 (36.3)	8 (26.6)		
**Education level, No. (%)**				3.9	0.14
Secondary education	11 (36.3)	8 (26.6)	9 (30)		
Diploma	9 (30)	13 (43.3)	14 (46.6)		
Academic	10 (33.3)	9 (30)	7 (23.3)		
**Overall quality of life, mean (SD)**	40.1 (3.6)	42.4 (4.7)	39 (3.1)	F = 4.88	0.3

Mean scores of QOL dimensions of the intervention groups were increased at the end of the study, then the mean differences in most of the QOL dimensions were tangibly positive in the intervention groups except for bodily pain. However, in the control group, the mean differences were negative or very low positive in the dimensions (Table 2). Also, as Table 2 shows, significant differences were observed between the mean differences of all QOL dimensions in the three groups (P < 0.05), except for the dimensions of emotional roles and bodily pain. Tukey post-Hoc test showed that except for the dimension of general health, the two intervention groups were not significantly different in other dimensions, and the observed significant differences were between the control group and the two intervention groups. However, a significant difference was observed between the lecture group and control group regarding general health (P = 0.012) (Table 2). 

**Table 2.** Comparing the Mean Differences^a ^of Quality of Life Dimensions in Patients With Hypertension at the Beginning, and the End of Study

Quality of Life Dimensions	Lecture Group, Mean (SD)	Educational Package Group, Mean (SD)	Control Group, Mean (SD)	Test Results, ANOVA, P value
**General health**	2.25 (0.1)	0.07 (0.01)	-1.70 (0.01)	0.04,Tukey (1 and 3): 0.01
**Social function**	9.81 (1.6)	8.22 (1.9)	5.40 (1.4)	0.01
**Physical function**	5.70 (1.2)	4.90 (0.9)	-1.40 (0.01)	0.003
**Emotional role**	0.7 (0.01)	8.60 (0.8)	-0.80 (0.02)	0. 21
**Physical role**	7.37 (1.8)	9.20 (2)	0.90 (0.2)	0.04
**Bodily pain**	-3.5 (0.02)	-2.1 (0.01)	0.20 (0.01)	0.06
**Power and energy**	9.5 (1.1)	0.10 (0.02)	1.10 (0.1)	0.002
**Mental health**	5.0 (0.9)	4.10 (1.1)	1.30 (0.6)	0.03
**Overall quality of life **	56.12 (9.7)	51.7 (6.9)	40.3 (4.5)	0.04

^a^ The mean differences defined by the mean scores of quality of life dimensions at the end of the study minus the mean scores of life quality dimensions at the beginning of study.

## 5. Discussion

Comparing the QOL in the three groups showed that there were significant differences in the QOL dimensions at the end of the study. These findings are consistent with the results of Juilliere et al. who studied the effect of therapeutic education in patients with chronic heart failure (20). It seems that both lecture and educational package could impact positively on most dimensions of QOL in patients with hypertension. Chiou and Chung have also reported that patient education using interactive multimedia could improve the knowledge, uncertainty, decision-making and overall QOL in patients with hypertension and end-stage renal disease (21). Berndt et al. in a review on the effect of patient education on QOL of patients with myocardial revascularization pointed that face to face education methods such as lectures may have the same effects in comparison with education methods together with indirect telephone follow-up (22). Similar results were reported in a study by Baraz-parjani et al., who studied the effect of self-care education in patients under dialysis (23). In another study, Stromberg et al. have studied the effects of self-care education on patients with cardiovascular disorders. They trained patient through lectures or a computer-based method and reported that lecture education was more effective than indirect education using a computer-based method (24). Moreover, Palumbo et al. studied the effect of educational booklets in patients with hypertension, and reported that face to face education was more effective than indirect education trough booklets (25). Educational booklets have some benefits such as being affordable, simple to use and clients’ ability to keep it with themselves, and referring to it at any time. However, it seems that patient education through lecture and face to face methods is generally more effective than indirect educations using pamphlets or computer-based methods. Perhaps lectures are more effective because educators have direct face to face interactions with clients. Such a direct interaction may deepen the effect of training. In the present study, two educational methods did not affect the dimensions of bodily pain and emotional roles. A study that performed by Matura et al. has also showed that education in patients with hypertension did not improve mental health and bodily pain and emotional roles (26). Nonetheless, the finding of the present study was inconsistent with the results of Moullec et al. who reported that self-management education could only increase emotional role of the QOL (27). However, Altuntas et al. have reported that education has improved all dimensions of QOL (28). The inconsistencies between different studies may be attributed to differences in educational methods, educational contents, mental and physical conditions of patients and the education settings. Generally, findings of this study showed that lecture and educational package can improve some dimensions of QOL in patients with hypertension including general health, social function, physical function, physical role, power and energy, and mental health. Although both methods were effective to some extent, selecting the best method depends both on the patients’ capabilities, and physical and human resources available in different settings. However, as pamphlets are cheap and easy to use, this may be used as an effective method for self-care education in health care settings in Iran, where the system is faced with nursing shortage. Finally, this study had some limitations. For instance, the patients may receive some informal information on their self-care from other recourses that was not under control of the researchers. 
